# Integrating Genomic Selection and Genome-Wide Association Study to Enhance Reproductive Traits in Thai Swamp Buffalo

**DOI:** 10.3390/ani15162333

**Published:** 2025-08-08

**Authors:** Rawinan Lomngam, Vibuntita Chankitisakul, Monchai Duangjinda, Wootichai Kenchaiwong, Kecha Kuha, Kritsanathon Sintala, Kulphat Pothikanit, Wuttigrai Boonkum

**Affiliations:** 1 Department of Animal Science, Faculty of Agriculture, Khon Kean University, Khon Kean 40002, Thailand; lo_rawinan@kkumail.com (R.L.); vibuch@kku.ac.th (V.C.); monchai@kku.ac.th (M.D.); 2 Network Center for Animal Breeding and Omics Research, Khon Kaen University, Khon Kaen 40002, Thailand; wootken@gmail.com; 3 Small Ruminant Research Unit, Faculty of Veterinary Science, Mahasarakham University, Mahasarakham 44000, Thailand; 4 Department of Animal Science, Faculty of Agriculture, Ubon Ratchathani Rajabhat University, Ubon Ratchathani 33000, Thailand; k.kuha3@gmail.com; 5 Department of Animal Science and Fisheries, Faculty of Sciences and Agricultural Technology, Rajamangala University of Technology Lanna, Nan 55000, Thailand; kitsanathon@rmutl.ac.th; 6 Buffalo Research and Development Group, Animal Breed Development Office, Department of Livestock Production, Bangkok 10400, Thailand; kulphatpothikanit@gmail.com

**Keywords:** age at first calving, calving interval, genetic parameter, candidate gene, SNP

## Abstract

Reproductive inefficiencies, such as delayed age at first calving (AFC) and a prolonged calving interval (CI), hinder the productivity of Thai swamp buffaloes. This study aimed to improve the genetic evaluation of these traits by integrating genomic selection (GS) and genome-wide association studies (GWASs). Reproductive records (n = 1034) and genotypes (n = 474) from swamp buffaloes across Thailand were analyzed. Variance components were estimated using pedigree data, and genomic predictions were performed via weighted single-step genomic best linear unbiased prediction. AFC heritability was moderate (0.36 and 0.45), whereas CI heritability was low (0.051 and 0.043). The positive genetic correlation ($r_{g}$ = 0.495 and 0.517) between AFC and CI suggested potential for the simultaneous genetic improvement of both traits through selection. The WssGBLUP method showed greater effectiveness, with the prediction accuracy increasing by up to 41% for CI and 28% for AFC when compared to the ABLUP method. The GWAS revealed 20 associated single-nucleotide polymorphisms (SNPs) on chromosomes 3, 4, 14, 15, and 25. Candidate genes *COLEC10*, *TNFRSF11B*, *PDZRN4*, and *MACROD2* were linked to immune function, hormonal regulation, and reproductive tissue development. Pleiotropic SNPs affecting both traits were identified, indicating shared genetic control mechanisms. These findings support the application of tailored GS programs to improve reproduction in swamp buffaloes under tropical smallholder systems.

## Simple Summary

Reproductive traits are critical for buffalo productivity and farm profitability. Genomic selection and genome-wide association studies were performed to identify key genetic markers and improve breeding value predictions. The integration of genomic data increased prediction accuracy. Several single-nucleotide polymorphisms (changes in the DNA at specific positions) and pleiotropic genes (genes that influence more than one trait) related to fertility were identified. These findings support the use of genetic data in buffalo breeding programs to enhance reproductive efficiency and productivity in smallholder farming systems under tropical conditions.

## 1. Introduction

Buffaloes (*Bubalus bubalis*) play a crucial role in the agricultural economies of South and Southeast Asia. They serve as vital sources of milk and meat, alongside their traditional use as draft animals. Their importance is underscored by their adaptability and productivity, both of which are essential for food security and rural prosperity [1,2,3]. Among the two major types—river and swamp buffalo—the Thai swamp buffalo represents a considerable indigenous resource with unique adaptive traits. In Thailand, these animals have historically been valued for their resilience in low-input systems, tolerance to tropical heat and parasites, and cultural and socioeconomic relevance, particularly among smallholder farmers [4,5].

However, as of 2024, their numbers had declined by 56% compared to those 20 years ago, reaching 1,815,901 animals [6,7]. Despite their strengths, their production efficiency remains below standard, particularly in terms of reproductive performance [8,9]. Similar findings have been reported in other countries, including India, Pakistan, and Nepal [10,11,12]. Key traits such as age at first calving (AFC) and calving interval (CI) directly influence the reproductive efficiency and lifetime productivity of female buffaloes [13]. Improving these traits is essential for enhancing overall herd fertility, reducing non-productive periods, and increasing economic returns to farmers.

Although reproductive efficiency is a cornerstone of sustainable buffalo production, swamp buffaloes are characterized by delayed puberty, long inter-CIs, and seasonal breeding behavior [14]. Under smallholder management, the AFC often exceeds 36 months, whereas CIs may surpass 500 days [8]. These inefficiencies result in fewer calves per lifetime, reducing milk and meat output. From an economic standpoint, prolonged AFC and CIs result in delayed returns on investment, increased maintenance costs, and lowered genetic progress due to longer generation intervals [15,16]. Socially, these constraints impact the livelihoods of rural households that rely on buffaloes for income, manure, draught power, and asset security [17,18]. Thus, the genetic improvement of reproductive traits such as AFC and CI has both developmental and economic significance.

Traditional breeding strategies in Thai swamp buffaloes face several constraints, including the absence of structured breeding programs, long generation intervals, limited pedigree recording, and a lack of performance data [9]. Phenotypic selection alone is insufficient for accelerating genetic gains in the complex reproductive traits. These traits often exhibit low heritability and are influenced by environmental, physiological, and management factors. Although advances in artificial insemination and reproductive technologies have benefited the dairy and beef cattle sectors, their adoption in swamp buffaloes remains limited. This is largely because of anatomical, behavioral, and logistical challenges. Consequently, genetic progress in reproductive performance has been slow.

Recent advances in genomic tools for livestock breeding have opened new avenues for improving traits with complex inheritance. For instance, genomic selection (GS) uses genome-wide single-nucleotide polymorphism (SNP) markers to determine genomic estimated breeding values (GEBVs). This approach has demonstrated considerable success in improving reproductive traits in dairy cattle, swine, and chicken [19,20,21,22]. Similarly, genome-wide association studies (GWASs) help to identify quantitative trait loci (QTLs) and candidate genes associated with reproductive traits, revealing their underlying genetic architecture [23,24]. The integration of GS and GWASs allows for both prediction and discovery, facilitating more accurate selection and deeper biological understanding.

Despite these advances, the application of GS and GWASs in swamp buffaloes—particularly in Thai populations—remains in its infancy. Existing studies have predominantly focused on river buffaloes, such as the Murrah and Nili-Ravi breeds, for which large-scale phenotypic and genotypic datasets are available [25,26,27]. In contrast, research on Thai swamp buffaloes has been constrained by small population sizes, limited genomic resources, and the absence of species-specific SNP arrays. Moreover, the lack of studies targeting the AFC and CI in swamp buffaloes represents a critical gap in the literature. Furthermore, the genetic correlations between these traits and other economically important attributes remain poorly understood, as does the extent of genetic variability within this indigenous population.

Several studies have begun to explore the potential of genomics in buffalo reproduction. For example, GWASs in Murrah buffaloes have allowed for the identification of significant SNPs associated with the AFC and CI on chromosomes harboring candidate genes involved in hormone regulation, ovarian development, and neuroendocrine pathways. Similarly, several candidate genes linked to fertility have been identified in riverine buffaloes [28,29]. However, whether these loci are relevant in swamp buffaloes remains uncertain due to differences in genetic background and adaptation. Moreover, no consistent evidence of pleiotropic genes or SNPs with major effects across multiple traits has been reported in swamp buffaloes. This inconsistency underscores the requirement for breed-specific investigations.

The urgency of this research is heightened by the need to improve livestock productivity under climate variability and resource constraints. As Thailand transitions toward sustainable agriculture and climate-resilient livestock systems, enhancing the reproductive performance of native breeds is a strategic priority. Genomic tools enable early selection, reduced generation intervals, and identification of animals that are both productive and adapted to local environments. Additionally, the availability of high-throughput genotyping platforms and advances in computational biology now make it feasible to apply GS and GWASs to local breeds previously excluded from genomic programs.

Given these considerations, the present study aimed to (i) estimate genetic parameters for AFC and CI in Thai swamp buffaloes using genomic information; (ii) evaluate the accuracy of genomic prediction models in these traits; and (iii) identify genomic regions and candidate genes associated with AFC and CI through a GWAS. This research seeks to establish foundational knowledge for implementing GS in Thai swamp buffalo breeding programs and contribute to the genetic improvement of reproductive efficiency in indigenous livestock in tropical production systems.

## 2. Materials and Methods

This study was conducted at Khon Kaen University and received ethical approval from the Institutional Animal Care and Use Committee (IACUC), in accordance with the Guidelines for the Ethics of Animal Experimentation established by the National Research Council of Thailand (Approval No. IACUC-KKU-24/67).

### 2.1. Data Collection

A total of 1034 records for reproductive traits—specifically, AFC and CI (the interval between the first and second calving)—were collected from Thai swamp buffaloes between 2019 and 2024. The pedigree file contained records spanning three generations and included a total of 2843 animals. Of these, 95.2% had known sire and dam information. These data were obtained from animal breeding research stations under the Thai Department of Livestock Development, a network of collaborating farmers across all regions of Thailand, and private buffalo farms. Genotype data were obtained from 474 Thai swamp buffaloes using the Axiom^®^ Buffalo 90K SNP Genotyping Array (Thermo Fisher Scientific, Santa Clara, CA, USA) and the GeneTitan™ system. Animals were selected for genotyping via stratified random sampling across high, medium, and low estimated breeding value (EBV) categories for both sexes. Quality control was applied to both animals and markers using the following criteria: a minor allele frequency of >5% and a call rate of ≥90% per marker. SNPs and animals failing to meet these thresholds were excluded. Additional exclusions comprised animals with duplicate genotypes and SNPs showing Mendelian conflicts. All quality control procedures and SNP analyses were performed using the BLUPF90+ version 2.56 software. After quality screening, 462 animals and 30,979 SNPs were retained for AFC and CI analyses. Summary statistics for these traits are presented in Table 1.

### 2.2. Genetic Parameter Estimation

Variance components for AFC and CI were estimated via pedigree and single-step genomic restricted maximum likelihood (REML) module using a bivariate animal model. The AIREMLF90 and BLUPF90+ programs [30] were used to estimate variance components and genetic parameters, such as heritability ${(h}^{2})$, genetic correlations $\left(r_{g}\right)$, and phenotypic correlations $\left(r_{p}\right)$. The bivariate animal model was specified as follows:

y = Xβ + Za + ε

where y is the vector corresponding to the observed values of AFC and CI; β is the vector of fixed effects, including herd–month–year of birth; a is the vector of random direct additive genetic effects assumed to be $a\sim N\left(0,A\sigma_{a}^{2}\right),$ where A is an additive genetic relationship matrix using pedigree information and $\sigma_{a}^{2}$ is the direct additive genetic variance; ε is the vector of random residual effects assumed to be $\varepsilon \sim N\left(0,I\sigma_{e}^{2}\right),$ where $\sigma_{e}^{2}$ is the residual variance; and X and Z are incidence matrices related to the vectors β and a, respectively.

### 2.3. Estimation of GEBVs

Genomic analyses were conducted using the weighted single-step genomic best linear unbiased prediction (WssGBLUP) method proposed by Wang et al. [31]. This approach integrates marker information by substituting the inverse pedigree relationship matrix ($A^{-1}$) in mixed model equations with $H^{-1}$ [32], as shown below:

$$H^{-1}\;=\;A^{-1}\;+\;\left[\begin{array}{cc} 0 & 0\\ 0 & {\tau G}^{-1}\;-\;{\omega A}_{22}^{-1} \end{array}\right]$$

where $A^{-1}$ is the inverse pedigree relationship matrix for all animals; $G^{-1}$ is the inverse genomic relationship matrix for genotyped animals; and $A_{22}^{-1}$ is the inverse pedigree relationship matrix. Weighting factors τ = 1.00 and ω = 0.50 were optimized to minimize bias during preliminary validation. The genomic relationship matrix (G) and SNP weights were constructed according to the method described by VanRaden [33], as shown below:

$$G\;=\;\frac{{ZDZ}^{\prime }}{2\sum_{i\;=\;1}^{N}p_{i}(1\;-\;p_{i})\;}=\;{ZDZ}^{\prime }\varphi \;$$

where ***Z*** denotes a matrix of allele counts adjusted for allele frequencies (coded as 0, 1, or 2 for genotypes aa, Aa, and AA, respectively); D is a diagonal matrix of SNP-specific weights; N is the total number of SNP markers; $p_{i}$ denotes the minor allele frequency for the ith SNP; and φ represents the global scaling factor derived from $\varphi \;=\;\frac{\sigma_{u}^{2}}{\sigma_{a}^{2}}\;=\;\frac{1}{2\sum_{i\;=\;1}^{N}p_{i}(1\;-\;p_{i})}$. The algorithm then proceeds as described by Wang et al. [31] and Chen et al. [34]. The accuracy of EBVs (${Acc}_{EBV}$) obtained from pedigree-based best linear unbiased prediction (ABLUP) and the accuracy of GEBVs (${Acc}_{GEBV}$) obtained from WssGBLUP were calculated and compared. The comparisons were based on the correlations (CORRs) between the breeding values (EBV and GEBV), the percentage of bias reduction (%Bias Reduction) calculated as $\frac{\left|{Avg}_{EBV}\;-\;{Avg}_{GEBV}\right|}{{\;|Avg}_{EBV}|}\;\times \;100$ [33,35,36], and the accuracy increase (%) calculated as $\frac{{Acc}_{GEBV}\;-\;{Acc}_{EBV}}{{Acc}_{EBV}}\;\times \;100$.

### 2.4. GWAS

The analyses were performed using WssGWAS. The same mixed model used in WssGBLUP was employed to estimate associations between quantitative traits and individual SNPs. SNP effects were derived through the iterative process described by Wang et al. [37] using the POSTGSF90 version 1.83 software [38]. The additive genomic breeding value vector ($\hat{u}$) was converted to SNP effects ($\hat{s}$) using their shared genomic variance $(\sigma_{u}^{2}$) in the following formula:

$$\hat{s}\;=\;{DZ}^{\prime }G^{-1}\hat{u}$$

The proportion of additive genetic variance explained by each 10-SNP window (to smooth out noise and account for LD) was calculated as follows:

$$\frac{Var\;({\hat{s}}_{j})}{{\hat{\sigma }}_{u}^{2}}\;\times \;100\%\;=\;\frac{Var\;(\sum_{k\;=\;j}^{j\;+\;9}z_{j}{\hat{s}}_{j})}{{\hat{\sigma }}_{u}^{2}}\;$$

where ${Var(\hat{s}}_{j})\;$ represents the estimate of segment variance for the window starting at the jth SNP (10 consecutive SNPs); ${\hat{\sigma }}_{u}^{2}$ denotes the total additive genetic variance; $z_{j}$ is the vector of allele counts for the jth SNP across all individuals; and ${\hat{s}}_{j}$ is the estimated effect of the jth SNP within the window [39]. The threshold for significant SNP variance was determined by selecting windows with high explained variance, accounting for at least 10% of the total additive genetic variance. Manhattan plots for these windows were generated using SAS Studio online version 3.81.

### 2.5. Identification of Candidate and Pleiotropic Genes

Potential candidate genes associated with AFC and CI were identified by locating genes within 50 kilobases (kb) of significant SNPs, based on previous studies [40,41]. Genes located in the same genomic region across one or two traits were classified as pleiotropic genes. Gene annotations were retrieved using the National Center for Biotechnology Information (NCBI) Genome Data Viewer (water buffalo (*B. bubalis*) genome; UMD 3.1 assembly as reference). Further functional information for identified genes was collected from the NCBI (https://www.ncbi.nlm.nih.gov/ accessed on 10 March 2025) and GeneCards (https://www.genecards.org/ accessed on 10 March 2025) platforms.

## 3. Results

### 3.1. Descriptive Statistics of Reproductive Traits

Descriptive statistics and genotype summary data for AFC and CI in Thai swamp buffalo are presented in Table 1. For AFC, the average was 46.31 months, with a standard deviation (SD) of 10.01 months. The trait exhibited substantial variability, ranging from a minimum of 32.00 months to a maximum of 66.33 months, corresponding to a coefficient of variation (CV) of 21.62%. For CI, the average was 520.87 days, with an SD of 103.68 days. The observed range was also wide, spanning 447.64–624.55 days and yielding a high CV of 19.91%.

### 3.2. Variance Components and Genetic Parameter Estimates

The variance components and genetic parameters for AFC and CI in Thai swamp buffaloes, estimated using both ABLUP and WssGBLUP, are presented in Table 2. Using the ABLUP method, the additive genetic variance ($\sigma_{a}^{2}$) was estimated at 72.27 for AFC and 6211.20 for CI, whereas the residual variances ($\sigma_{e}^{2}$) were 130.37 and 114510.00, respectively. The corresponding heritability estimates ($h^{2}\;\pm \;SE$) were moderate for AFC (0.36 ± 0.027) but low for CI (0.051 ± 0.004). The additive genetic variances increased to 88.91 for AFC and slightly decreased to 5223.90 for CI using the WssGBLUP model. The residual variances were similar to those observed using ABLUP ($\sigma_{e}^{2}$ = 109.03 for AFC and 114920.00 for CI). Incorporating genomic information via WssGBLUP resulted in an increased heritability estimate for AFC (0.45 ± 0.026), whereas the estimate for CI remained unchanged (0.043 ± 0.003). Genetic correlations ($r_{g}$) between AFC and CI were positive across both models: 0.495 for ABLUP and 0.517 for WssGBLUP. This suggests that animals with earlier calving tend to have shorter CIs. Correspondingly, phenotypic correlations ($r_{p}$) were also positive (0.467 and 0.473, respectively).

### 3.3. Comparison of EBVs and GEBVs for Reproductive Traits

The comparison between EBVs and GEBVs for AFC and CI in Thai swamp buffaloes is presented in Table 3. This analysis was based on correlation coefficients between the accuracies of EBVs and GEBVs, percentage bias reduction, and percentage increases in accuracy across various datasets. For AFC, the correlation between GEBV and EBV accuracies remained consistently high across all subsets, ranging from 0.968 (top 20% of all animals) to 0.999 (dam dataset). Incorporating genomic information led to notable improvements in prediction accuracy, with increases ranging from 12.48% (the top 20% of bulls) to 28.34% (the full dataset). Notably, the dam dataset achieved one of the highest accuracy gains (26.61%) with the highest correlation coefficient (r = 0.999), emphasizing the value of female genotyping in genomic evaluations. The bias reduction was also substantial, particularly in the top 20% of all animals (−35.33%) and the dam dataset (−30.14%).

Similar trends were observed for CI. Correlation coefficients ranged from 0.977 (the top 20% of all animals) to 0.999 (the dam dataset), underscoring the consistency of GEBVs with traditional EBVs. The highest accuracy improvement was seen in the full dataset (41.07%), followed by dams (39.20%) and the top 20% of all animals (39.57%). The bias reduction was moderate but consistent, ranging from −1.34% to −14.63%, with the highest reduction recorded in the dam dataset.

### 3.4. SNP Effects and Genomic Regions Associated with Reproductive Traits

**Figure 1:**
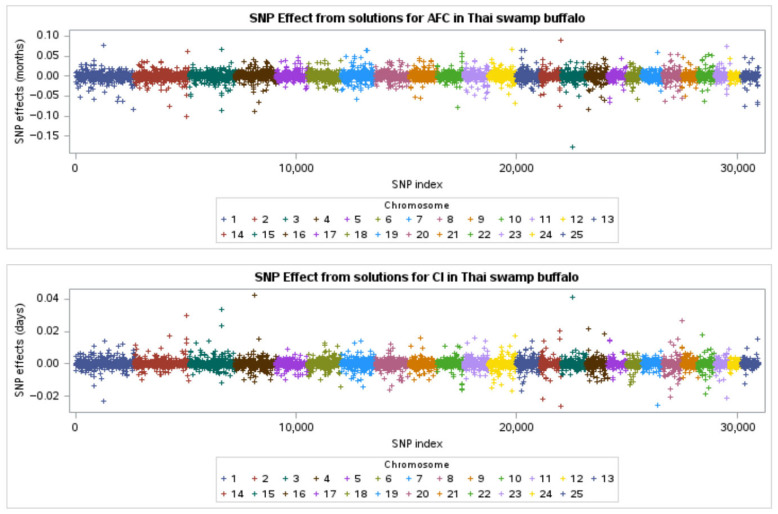
Single-nucleotide polymorphism (SNP) effects derived from GEBVs for age at first calving (AFC) and calving interval (CI) in Thai swamp buffaloes.

**Figure 2:**
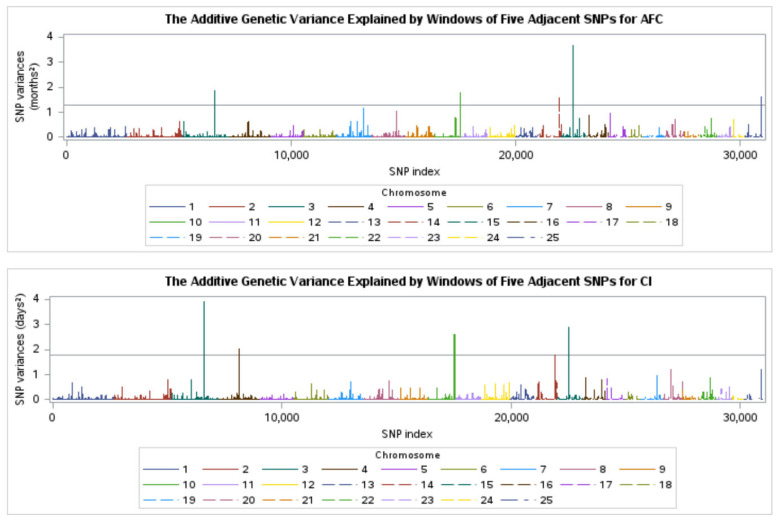
Manhattan plots of the additive genetic variance explained by windows of five adjacent SNPs for AFC and CI in Thai swamp buffaloes.

The SNP effects derived from GEBVs for AFC and CI in Thai swamp buffaloes are shown in Figure 1. The distribution of SNP effects across the genome varied between traits, reflecting the polygenic nature of reproductive performance. For AFC, a subset of SNPs exerted relatively large positive or negative effects. In contrast, the SNP effects for CI were more evenly distributed. Manhattan plots were generated using a sliding window of five adjacent SNPs to assess the proportion of additive genetic variance explained to further characterize the genomic architecture (Figure 2). For AFC, significant genomic regions were identified on chromosomes BTA3, BTA7, and BTA14. These loci explained a notable proportion of the total additive variance and may be associated with age at reproductive onset. Similarly, for CI, prominent peaks were observed on BTA5, BTA10, and BTA17, suggesting their involvement in postpartum reproductive physiology and calving cycle regulation.

### 3.5. WssGWAS

**Figure 3:**
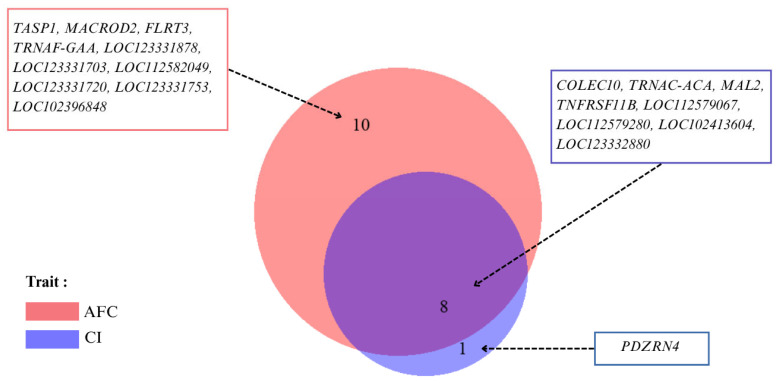
Pleiotropic genes identified for AFC and CI in Thai swamp buffaloes.

The WssGWAS identified a set of pleiotropic genes significantly associated with both AFC and CI in Thai swamp buffaloes (Figure 3). These genes were distributed across multiple chromosomes and contributed jointly to the genetic control of reproductive traits. This suggests that shared biological mechanisms may underlie both sexual maturity and postpartum fertility. Notably, some pleiotropic loci overlapped with regions showing high additive genetic variance in earlier GWAS results, reinforcing their relevance to reproductive performance.

A total of 20 SNPs exceeded the genome-wide significance threshold for association with AFC and CI (Table 4), with major loci concentrated on chromosomes 3, 4, 14, 15, and 25. The most prominent associations were observed on chromosome 15, where SNPs such as AX-85068166, AX-85072819, AX-85075636, and AX-85129152 were significantly associated with both reproductive traits. Notably, these markers were located near genes implicated in immune response and developmental processes, including collectin subfamily member 10 (*COLEC10*), TNF receptor superfamily member 11b (*TNFRSF11B*), and *TRNAC-ACA*. These genes are involved in neural crest migration, osteoclastogenesis, and translational fidelity, respectively, highlighting their potential regulatory roles in reproductive physiology.

In addition, non-coding RNA (ncRNA) regions such as *LOC112579067*, *LOC102413604*, and *LOC112579280* were found near or overlapping significant SNPs. These loci are predicted to function as molecular scaffolds involved in chromatin remodeling and transcriptional regulation, supporting their possible epigenetic roles in modulating reproductive traits. On chromosome 4, a cluster of SNPs—including AX-85081751, AX-85099246, and AX-85129182—was mapped onto the *PDZRN4* gene associated exclusively with CI. This gene encodes a PDZ domain-containing protein involved in protein degradation, cell signaling, and spermatogenesis. It is repeated across multiple SNPs, indicating its regulatory role in postpartum reproductive recovery.

On chromosomes 3 and 25, significant associations with AFC were found at AX-85087929 and AX-85170264, located near long ncRNA (lncRNA), such as *LOC123332880* and *LOC123331878*. These lncRNAs have been implicated in post-transcriptional regulation and RNA stability. The functional annotations of these loci suggest they may influence ovarian development and hormone signaling pathways critical for the initiation of reproductive maturity.

Additionally, SNPs AX-85048696 and AX-85123888 on chromosome 14 mapped onto *TASP1* and *MACROD2*, respectively. These genes are known to regulate embryonic development, genome stability, and cell proliferation. Their association with AFC implies a role in folliculogenesis or uterine preparation.

## 4. Discussion

This study integrated WssGBLUP with a GWAS to enhance the prediction accuracy of key reproductive traits—namely AFC and CI—in Thai swamp buffaloes. The combination of GS and GWAS provided complementary insights into the genetic architecture of these complex traits and identified candidate loci, offering substantial benefits for breeding programs aimed at improving reproductive efficiency. By leveraging genomic data from over 30,000 SNPs and reproductive records from more than 1000 animals, this study provided a comprehensive analysis of traits that are not only economically important but also critical for herd productivity and sustainability in low-input systems.

The estimated heritability for AFC using both traditional ABLUP (0.36) and genomic WssGBLUP (0.45) indicated moderate genetic control. This aligns with previous reports in riverine buffaloes (such as Murrah and Nili-Ravi’s studies) and suggests that AFC is amenable to improvement through selection [42,43]. The increased heritability under WssGBLUP underscores the value of incorporating genomic data to enhance trait predictability, particularly for traits with moderate polygenic control. Conversely, CI exhibited a remarkably low heritability (0.051 and 0.043) across both models, reflecting its high environmental sensitivity. Similar findings have been reported in previous studies [44,45]. This suggests that non-genetic factors such as nutrition, management, health, estrus detection, and seasonal breeding behavior play dominant roles in CI expression [46,47]. Thus, improvements in CI will require a combined approach of genetic selection and management interventions.

The estimated genetic correlations between AFC and CI in swamp buffalo were positive and moderate in both methods, indicating a moderate synergistic genetic relationship between the two reproductive traits. Similar positive associations have been reported in cattle [48,49] and both swamp and river buffaloes [15,43,44,50], highlighting the importance of employing multivariate genetic selection. Biologically, this positive correlation suggests that animals genetically predisposed to calve later for the first time are also more likely to experience longer intervals between subsequent calvings. Additionally, the delayed age at first calving (AFC) and extended calving intervals (CIs) in buffaloes can be attributed to several physiological and developmental factors such as suboptimal endocrine function and insufficient body conditions, which can hinder the resumption of ovarian cyclicity postpartum [51,52]. Understanding these relationships is crucial for improving reproductive efficiency in buffalo farming. Additionally, animals with inherently late AFC may carry genetic factors affecting reproductive hormone regulation, uterine involution, or ovarian follicular development [53], which can compromise subsequent reproductive efficiency. Finally, although the genetic correlation values in this study were slightly higher than those previously reported, we followed a standardized procedure for verifying pedigree, phenotypic, and SNP records to ensure the accuracy of the genetic parameter estimates.

The integration of genomic information using WssGBLUP substantially improved the accuracy of EBVs for both traits. For AFC, genomic prediction improved by up to 28.3% in the full dataset and by 26.6% in dams. For CI, the accuracy improvement was even more pronounced, with a 41.1% increase in the full dataset and over 39% in dams. These improvements are consistent with other reports in livestock species, where genomic evaluations outperform pedigree-based models [21,54]. Additionally, the dam subset demonstrated the highest prediction accuracy and bias reduction, emphasizing the value of incorporating female genotype data in genomic evaluations, particularly for maternally influenced traits such as AFC. Although male-based evaluations traditionally dominate breeding schemes due to the accessibility of semen, this study highlights the importance of a more balanced selection strategy incorporating both sexes. In the context of Thai swamp buffalo breeding, where pedigree information is often incomplete or unreliable, GS offers a robust alternative. Early identification of superior animals using GEBVs can shorten generation intervals, accelerate genetic gain, and support the sustainable intensification of buffalo production systems.

The GWAS identified several significant SNPs on chromosomes 3, 4, 14, 15, and 25, regions previously linked to reproductive traits in buffaloes and cattle [45,55,56]. The polygenic distribution of SNP effects, along with the presence of pleiotropic loci, highlights the complexity of AFC and CI and suggests the presence of shared regulatory mechanisms [45]. Key candidate genes identified included *COLEC10*, which is involved in embryonic development and immune response via the lectin pathway [57]; *TRNAC-ACA*, a tRNA gene critical for protein translation and cellular growth processes [28]; and *TNFRSF11B*, which is known for regulating osteoclastogenesis and reproductive tissue remodeling via the NF-κB signaling pathway [58,59]. The recurrent identification of *MACROD2* in association with AFC is also notable. This gene has been implicated in chromatin remodeling, DNA repair, and reproductive cell cycle regulation [60]. In addition, *PDZRN4*, identified exclusively for CI on BTA4, is involved in spermatogenesis, cell signaling, and immune regulation, suggesting a central role in postpartum fertility and uterine recovery [61,62].

Equally significant is the presence of regulatory ncRNA and lncRNA, such as *LOC123331878* and *LOC102413604*, near major SNPs. These regions likely contribute to the transcriptional regulation of reproductive pathways via chromatin interactions or epigenetic control, echoing trends reported in livestock fertility studies [63,64]. The identification of these genes supports the functional hypotheses regarding reproductive regulation in buffalo, indicating a potential for marker-assisted selection and future validation via transcriptomic and epigenomic approaches.

This study represents one of the most comprehensive attempts to apply GS and a GWAS in Thai swamp buffaloes. Previous studies have largely focused on river buffalo breeds, owing to their commercial prominence and the availability of large-scale datasets [27,65]. In contrast, swamp buffaloes have received limited genomic attention due to their declining population, the fragmented breeding infrastructure, and a lack of species-specific SNP chips. The development of a population-specific genomic reference using the Axiom^®^ 90K Buffalo Genotyping Array in this study marks an important advance. It enables the identification of breed-specific QTLs and facilitates the practical implementation of GS in Thai breeding programs.

Furthermore, predicting reproductive potential early in life—that is, prior to first calving—can help to reduce reproductive wastage and improve animal replacement strategies. The economic implications of improving AFC and CI are considerable. Earlier calving translates to quicker returns on investment, while shorter CIs increase the number of calves produced over a lifetime, enhancing both productivity and genetic progress. At a national level, improved reproductive efficiency in swamp buffaloes could revitalize the declining buffalo sector in Thailand, contributing to food security, rural economic development, and the conservation of indigenous genetic resources.

While this study provides strong evidence for the utility of GS and GWAS in reproductive improvement, several limitations warrant attention. First, the persistently low heritability of CI indicates limited genetic gain from selection alone. Therefore, genetic approaches must be complemented by reproductive management strategies, such as estrus synchronization, better nutrition, and improved healthcare. Second, the sample size of genotyped animals—although the largest to date in research on the Thai swamp buffalo—limited the detection of rare variants and QTLs with small effects. Increasing the number of genotyped animals and expanding the reference population would enhance the GWAS resolution and improve GEBV accuracy. Moreover, in this study, the low level of polymorphisms observed with the Axiom^®^ Buffalo 90K SNP Genotyping Array (Thermo Fisher Scientific, Santa Clara, CA, USA) in Thai swamp buffalo populations may have reduced the power to detect significant associations in the GWAS. This limitation likely stems from the fact that the array was originally designed based on river buffalo genomes, which may not adequately capture the genetic diversity present in swamp buffaloes. Future studies may benefit from the use of custom genotyping arrays or whole-genome sequencing-based platforms that are specifically tailored to swamp buffalo populations. Finally, the use of multi-trait models incorporating both reproductive and production traits—including growth, milk yield, and disease resistance—could improve selection efficiency. The inclusion of resilience traits, such as heat tolerance, would align buffalo breeding with climate-smart agriculture goals.

## 5. Conclusions

This study demonstrated the effectiveness of integrating genomic prediction with association mapping to elucidate the complex genetic architecture of reproductive traits in swamp buffaloes. The results support the adoption of GS programs targeting AFC and CI to shorten generation intervals and improve reproductive efficiency. Given the low heritability of CI, however, achieving meaningful genetic gains will require the concurrent improvement of management practices and health interventions. Future research should prioritize the functional annotation of significant loci, validation in independent populations, and development of multi-trait GS indices that encompass fertility, growth, and resilience traits.

## Figures and Tables

**Table 1:** Descriptive statistics and genotype data for reproductive traits in Thai swamp buffaloes.

Traits	Mean	SD	Min.	Max.	%CV
AFC (months)	46.31	10.01	32.00	66.33	21.62
CI (days)	520.87	103.68	447.64	624.55	19.91

AFC = age at first calving; CI = calving interval at first parity; Mean = average of traits; SD = standard deviation; Min. = minimum; Max. = maximum; CV = coefficient of variation.

**Table 2:** Comparison of variance components and genetic parameters for reproductive traits in Thai swamp buffaloes using pedigree-based best linear unbiased prediction (ABLUP) and weighted single-step genomic best linear unbiased prediction (WssGBLUP).

Methods	ABLUP		WssGBLUP	
Parameters	AFC	CI	AFC	CI
$\sigma_{a}^{2}$	72.27	6211.20	88.91	5223.90
$\sigma_{e}^{2}$	130.37	114,510.00	109.03	114,920.00
$h^{2}\;\pm \;SE$	0.36 (0.027)	0.051 (0.004)	0.45 (0.026)	0.043 (0.003)
$r_{g}$	0.495		0.517	
$r_{p}$	0.467		0.473	

AFC = age at first calving; CI = calving interval; $\sigma_{a}^{2}$ = additive variance; $\sigma_{e}^{2}$ = residual variance; $h^{2}$ = heritability; $r_{g}$ = genetic correlation; $r_{p}$ = phenotypic correlation.

**Table 3:** Comparison between estimated breeding values (EBVs) and genomic estimated breeding values (GEBVs) for reproductive traits in Thai swamp buffaloes based on correlation, bias reduction, and accuracy.

Dataset	CORR	Bias Reduction (%)	Accuracy Increase (%)
AFC			
All animal datasets	0.984	−12.939	28.342
Bull dataset	0.991	−21.538	25.858
Dam dataset	0.999	−30.140	26.606
Top 20% of all animals	0.968	−35.334	26.076
Top 20% of bulls	0.989	−12.950	12.481
Top 20% of dams	0.994	−2.196	25.949
CI			
All animal datasets	0.984	−1.339	41.070
Bull dataset	0.991	−5.945	38.224
Dam dataset	0.999	−14.627	39.196
Top 20% of all animals	0.977	−12.105	39.571
Top 20% of bulls	0.996	−2.533	23.576
Top 20% of dams	0.988	−7.684	39.057

AFC = age at first calving; CI = calving interval; CORR = correlation.

**Table 4:** Significant SNPs associated with AFC and CI in Thai swamp buffaloes.

No.	SNP Name	SNP Variance	Chromosome	Location (bp)	Gene	Size (bp)	Distance to SNP (bp)	Putative Function	Associated Traits
1	AX-85068166	3.653 2.884	15	36,985,581	*COLEC10*	46,505	−5651	Innate immune response via lectin complement pathway; involved in embryonic development (neural crest cell migration).	AFC, CI
					*LOC112579067*	62,540	20,530	Likely acts as a regulatory ncRNA involved in gene expression modulation via chromatin interactions, transcriptional regulation, or as a molecular scaffold; may influence developmental and physiological pathways relevant to reproductive traits.	
2	AX-85072819	3.653 2.885	15	37,187,614	*TRNAC-ACA*	73	4031	Involved in the delivery of amino acid cysteine to the ribosome during protein translation; plays a crucial role in maintaining translational fidelity and efficiency.	AFC, CI
					*MAL2*	33,379	24,124	Encodes a multispan transmembrane proteolipid involved in lipid raft-mediated transcytosis in polarized epithelial cells; may regulate membrane protein sorting and vesicular transport.	
3	AX-85075636	3.652 2.886	15	37,227,004	*TRNAC-ACA*	73	43,421	Involved in the delivery of amino acid cysteine to the ribosome during protein translation; plays a crucial role in maintaining translational fidelity and efficiency.	AFC, CI
4	AX-85129152	3.652 2.880	15	36,861,303	*TNFRSF11B*	28,196	9794	Acts as a secreted decoy receptor for RANKL and TRAIL; inhibits osteoclastogenesis and bone resorption and regulates bone remodeling; modulates NF-κB-mediated immune signaling and may prevent vascular calcification.	AFC, CI
					*LOC112579280*	135	17,407	Acts as an H/ACA box snoRNA guiding pseudouridylation of rRNA and potentially other RNAs; may regulate RNA processing, ribosome function, and cellular proliferation.	
					*LOC102413604*	347,952	14,148	Likely functions as a regulatory ncRNA involved in modulation of gene expression via chromatin interactions or transcription regulation; may act as a molecular scaffold and influence developmental and reproductive pathways.	
					*LOC112579067*	62,540	−41,209	Likely acts as a regulatory ncRNA involved in gene expression modulation via chromatin interactions, transcriptional regulation, or as a molecular scaffold; may influence developmental and physiological pathways relevant to reproductive traits.	
5	AX-85102487 AX-85149742	3.651 2.880	15	36,559,391	*LOC102413604*	347,952	On target	Likely functions as a regulatory ncRNA involved in modulation of gene expression through chromatin interactions or transcription regulation; may act as molecular scaffold and influence developmental and reproductive pathways.	AFC, CI
6	AX-85087929 AX-85133105	1.862 3.896	3	130,355,532	*LOC123332880*	76,994	On target	Likely functions as a regulatory lncRNA involved in transcriptional and epigenetic regulation through chromatin remodeling, enhancer–promoter interactions, splicing, or acting as a molecular scaffold; may influence reproductive developmental pathways.	AFC, CI
7	AX-85170264	1.625	25	135,219,084	*LOC123331878*	95,019	On target	Likely functions as a regulatory lncRNA implicated in transcriptional or post-transcriptional regulation via chromatin remodeling, RNA splicing, RNA stabilization, or scaffold formation; potentially influences genes involved in reproductive development and physiological pathways.	AFC
8	AX-85125727 AX-85182553	1.621	25	135,311,866	*LOC123331703*	1118	−6735	May function as a processed pseudogene derived from MORF4L1; could act as a regulatory RNA influencing expression of the MORF/MRG gene family involved in chromatin remodeling, cell proliferation, senescence, and transcriptional regulation.	AFC
					*LOC112582049*	2037	−8970	Likely acts as a regulatory pseudogene derived from MORF4L1; may produce ncRNAs that serve as sponges for microRNAs or siRNA precursors; modulates expression of MORF/MRG chromatin remodeling complex, influencing cell proliferation, senescence, and transcriptional control.	
					*LOC123331720*	114,704	−17,294	Likely encodes a novel protein with potential enzyme or binding functions; may participate in intracellular signaling or protein complex formation; predicted involvement in developmental or reproductive pathways.	
					*LOC123331753*	7170	17,530	Likely functions as a regulatory lncRNA involved in transcriptional and post-transcriptional regulation (via chromatin remodeling, splicing, RNA stabilization, or molecular scaffolding); may influence reproductive pathway genes.	
					*LOC123331878*	95,019	38,369	Likely functions as a regulatory lncRNA implicated in transcriptional or post-transcriptional regulation; may act via chromatin remodeling, RNA splicing, RNA stabilization, or scaffold formation, potentially influencing genes involved in reproductive development and physiological pathways.	
9	AX-85048696 AX-85138148	1.607	14	76,488,914	*TASP1*	278,804	−19,341	Encodes a conserved threonine endopeptidase that proteolytically cleaves transcriptional regulators such as MLL1/2 and TFIIA, essential for *HOX* gene expression, embryonic development, hematopoietic stem cell maintenance, craniofacial morphogenesis, and cell cycle progression.	AFC
10	AX-85123888	1.606	14	76,079,203	*MACROD2*	2,318,987	On target	Catalyzes removal of mono-ADP-ribose from proteins, regulating DNA damage repair, chromatin remodeling, and Wnt/β-catenin signaling; implicated in cell proliferation, genome stabilization, and developmental processes.	AFC
					*LOC102396848*	835	−49,655	Likely acts as a regulatory pseudogene derived from SMIM20; may produce RNA transcripts functioning as ceRNA/miRNA sponges or antisense regulators, modulating SMIM20 expression involved in mitochondrial cytochrome c oxidase assembly and reproductive peptide (phoenixin) production influencing ovarian follicle growth and GnRH signaling.	
11	AX-85116617	1.565	14	75,859,588	*MACROD2*	2,318,987	On target	Catalyzes removal of mono-ADP-ribose from proteins, regulating DNA damage repair, chromatin remodeling, and Wnt/β-catenin signaling; implicated in cell proliferation, genome stabilization, and developmental processes.	AFC
					*FLRT3*	12,568	On target	Mediates cell–cell adhesion and receptor signaling; modulates FGF-ERK and VEGF-dependent pathways; involved in embryonic morphogenesis, neuronal guidance, angiogenesis, and tissue polarity.	
12	AX-85072646	1.562	25	135,219,084	*LOC123331878*	95,019	On target	Likely functions as a regulatory lncRNA implicated in transcriptional or post-transcriptional regulation; may act through chromatin remodeling, RNA splicing, RNA stabilization, or scaffold formation, potentially influencing genes involved in reproductive development and physiological pathways.	AFC
13	AX-85089841	1.531	14	75,807,197	*MACROD2*	2,318,987	On target	Catalyzes removal of mono-ADP-ribose from proteins, regulating DNA damage repair, chromatin remodeling, and Wnt/β-catenin signaling; implicated in cell proliferation, genome stabilization, and developmental processes.	AFC
14	AX-85071095 AX-85167220	1.518	25	135,188,395	*LOC123331878*	95,019	On target	Likely functions as a regulatory lncRNA implicated in transcriptional or post-transcriptional regulation; may act via chromatin remodeling, RNA splicing, RNA stabilization, or scaffold formation, potentially influencing genes involved in reproductive development and physiological pathways.	AFC
15	AX-85048217	1.308	3	130,427,805	*LOC123332880*	76,994	On target	Likely functions as a regulatory lncRNA involved in transcriptional and epigenetic regulation via chromatin remodeling, enhancer–promoter interactions, splicing, or acting as a molecular scaffold; may influence reproductive developmental pathways.	AFC
16	AX-85075413	1.308	3	130,503,159	*TRNAF-GAA*	73	16,083	Delivers phenylalanine to the ribosome during protein translation; essential for translational fidelity and efficiency.	AFC
17	AX-85129182 AX-85187994	2.030 2.029	4	80,876,599	*PDZRN4*	437,151	On target	Functions as a tumor suppressor and regulates cell proliferation and protein degradation pathways; involved in neuronal/neural crest development, spermatogenesis, and immune signaling via PDZ domain-mediated scaffold interactions.	CI
18	AX-85099246	2.028	4	81,019,718	*PDZRN4*	437,151	On target	Functions as a tumor suppressor, regulates cell proliferation and protein degradation pathways; involved in neuronal/neural crest development, spermatogenesis, and immune signaling via PDZ domain-mediated scaffold interactions.	CI
19	AX-85081751	2.017	4	80,809,832	*PDZRN4*	437,151	On target	Functions as a tumor suppressor and regulates cell proliferation and protein degradation pathways; involved in neuronal/neural crest development, spermatogenesis, and immune signaling via PDZ domain-mediated scaffold interactions.	CI
20	AX-85052779	1.950	4	80,872,538	*PDZRN4*	437,151	On target	Functions as a tumor suppressor and regulates cell proliferation and protein degradation pathways; involved in neuronal/neural crest development, spermatogenesis, and immune signaling via PDZ domain–mediated scaffold interactions.	CI

*COLEC10* = collectin subfamily member 10; *TRNAC-ACA* = transfer RNA cysteine (anticodon ACA); *MAL2* = Mal, T cell differentiation protein 2; *TNFRSF11B* = TNF receptor superfamily member 11b; *TASP1* = taspase 1; *MACROD2* = mono-ADP ribosylhydrolase 2; *FLRT3* = fibronectin leucine-rich transmembrane protein 3; *TRNAF-GAA* = transfer RNA phenylalanine (anticodon GAA); *PDZRN4* = PDZ domain-containing ring finger 4; *LOC112579067* = uncharacterized *LOC112579067*; *LOC112579280* = small nucleolar RNA *LOC112579280* (SNORA42/SNORA80 family); *LOC102413604* = uncharacterized *LOC102413604*; *LOC123332880* = uncharacterized *LOC123332880*; *LOC123331878* = uncharacterized *LOC123331878*; *LOC123331703* = mortality factor 4-like protein 1; *LOC112582049* = mortality factor 4-like protein 1; *LOC123331720* = uncharacterized *LOC123331720*; *LOC123331753* = uncharacterized *LOC123331753*; *LOC102396848* = small integral membrane protein 20-like.

## Data Availability

The original contributions presented in this study are included in this article; further inquiries can be directed to the corresponding author.
